# Assessment of food safety knowledge, attitudes, and self-reported practices among secondary school students in Bangladesh: A geographic information system-based cross-sectional study

**DOI:** 10.1371/journal.pgph.0007173

**Published:** 2026-09-10

**Authors:** Md. Nazrul Islam, Sumaia Tasnim, Sultan Mahmud Imran, Nitai Roy

**Affiliations:** 1 Department of Post Harvest Technology, Faculty of Nutrition and Food Science, Patuakhali Science and Technology University, Patuakhali, Bangladesh; 2 Department of Community Health and Hygiene, Faculty of Nutrition and Food Science, Patuakhali Science and Technology University, Patuakhali, Bangladesh; 3 Department of Biochemistry and Molecular Biology, Faculty of Nutrition and Food Science, Patuakhali Science and Technology University, Patuakhali, Bangladesh; PLOS: Public Library of Science, UNITED STATES OF AMERICA

## Abstract

Foodborne diseases are a significant public health problem in Bangladesh, particularly among school students with unsafe handling and consumption of food is a common practice. Therefore, this study assessed food safety knowledge, attitudes, and practices (KAP) among secondary school students across Bangladesh and examined socio-demographic predictors and division-wise variations in these areas. This cross-sectional study was conducted from 10 January to 25 May 2024 in eight districts covering all administrative divisions of Bangladesh. Using a multistage sampling method, 48 secondary schools were chosen, and 2,226 students aged 12–17 years and enrolled in grades 9 and 10 (the secondary education level in Bangladesh) participated in the study (94.4% response rate). A structured questionnaire was used to collect data on socio-demographics and the food safety KAP. Descriptive statistics and non-parametric tests were conducted using SPSS 26.0, and the division-wise variations were explored using ArcGIS 10.8. The mean knowledge score obtained was 5.04/10 (50.4%), indicating moderate knowledge with respect to personal hygiene and considerable gaps in handling expired foods and food storage practices. The average attitude was 24.56/50 (49.1%), indicating borderline positive attitudes with common misconceptions about food additives and expired foods. Food safety practices were low, with a mean score of 19.92/50 (39.8%), indicating low adherence to appropriate reheating, storage, and hygienic practices. Gender, academic class, parents’ education, income, and participation in food preparation were the major predictors, according to the regression models. There were variations across divisions, with Rangpur, Sylhet, and Mymensingh performing better in the KAP domains. Secondary school students in Bangladesh have moderate knowledge with borderline attitudes and poor food safety practices. Knowledge did not correlate with behavioral performance; however, attitudes strongly predicted practice. Personalized and targeted division-specific food safety education programs should be promoted to enhance adolescents’ food safety behaviors and mitigate their potential liability in unnecessary future outbreaks.

## Introduction

Food safety is universally acknowledged as an important and enduring worldwide public health problem that causes a great deal of human suffering, with serious economic implications for society, healthcare systems, and national productivity. Consuming contaminated food results in an alarming toll of foodborne illnesses, affecting millions at every socioeconomic level. The average figure of 600 million people per year, which accounts for almost one person in ten worldwide [1], is affected by diseases through contaminated food. With this colossal morbidity, the disease also carries a serious mortality risk, with approximately 420,000 estimated deaths globally every year [1]. The magnitude of this disaster highlights that food safety is an integral part of global public health and cannot be negotiated.

The disproportionate burden of foodborne diseases is the highest among those who are already vulnerable. Tragically, the burden of this disease is especially severe in children less than five years old, with 125,000 deaths annually [1]. This makes them vulnerable because of their immature immunity and sometimes increased hazards in terms of food processing or consumption environments [2]. In addition to the preschool age group, older children and other at-risk populations, including children, the elderly, pregnant women, and immunocompromised individuals, in particular, are at significant risk of foodborne disease [3,4]. This increase in foodborne diseases, directly linked to contaminated or unsafe foods, has therefore succeeded in consolidating improvements in the area of food safety as an increasingly important issue on the global health agenda [5]. This is supported by the consistent pattern of findings across various global health studies [6,7].

The etiology of foodborne diseases is intricate and multidimensional, involving a complex interplay of biological, chemical, and behavioral risk factors [8,9]. The most common causes of disease outbreaks in developing countries primarily emanate from entrenched systemic and infrastructural gaps [10,11]. These include inadequate infrastructure for processing and storage of food; the application of contaminated water during soaking, washing, and cooking processes in households; lack of good personal and community hygiene behavior practices; and poor understanding of the basic principles of safe food handling [10] which are predominant in commercial establishments as well as at home [11]. The lack of consumer knowledge, most notably among susceptible and autonomous populations such as children and adolescents, who tend to make their own food choices outside the home more frequently results particularly in a higher risk of acquiring foodborne diseases [12,13]. Even more disturbingly, on a few occasions, foodborne outbreaks were identified as emanating directly from the schools; thus, school settings and spaces educators like to think of as safe zones for health and well-being can also be quite risky for transmission [14,15]. The symptom onset delay is sufficiently long, and there are many risk factors (including cross-contamination, cooking temperature control, and basic personal hygiene by food handlers). Therefore, an integrated approach that encompasses legislation, infrastructure, and important education is required to lower these risks.

Global public health risks are particularly enhanced in Low- and Middle-Income Countries (LMICs), where resource constraints and large population densities augment exposure to food safety hazards [16–18]. Most LMICs, such as Bangladesh, face a range of frequent and severe food safety challenges that pervade an entire (often fragmented) food supply chain [13]. This ongoing problem is further intensified by issues related to national growth processes, such as excessive urbanization and ever-growing population complements [19,20]. These demographic and social transformations will ferment growing consumer demand for convenient, easily eaten foods, including those sold on the street and convenience meals. The large number of unstructured street food vendors throughout the country operating with little to no regulatory constraints and minimal sanitation also represent a significant and persistent threat to the safe handling and supply of foods [21,22].

The effects of these systemic food safety lapses in Bangladesh amount to national public health emergencies. It has been reported that 30 million people in Bangladesh are victims of foodborne diseases annually [23]. This undifferentiated, ubiquitous, and economically devastating set of diseases can be largely attributed to high population density, Water, Sanitation & Hygiene (WASH) status in a fragile condition, and consistently poor infrastructure for safe water supplies and waste management [24]. Among the foodborne diseases that have been widely reported in Bangladesh are severe diarrheal disease, enteric fever, and hepatitis (Food and Agricultural Organization of the United Nations) [25]. National surveillance data vividly highlight the enormity of the problem, picking up over 30,000 cases of enteric fever and 500 acute hepatitis cases in 2015 alone; figures that are generally acknowledged to represent a fraction of the actual hidden burden (Institute of Epidemiology, Disease Control and Research [26,27]. The emergency response to this crisis requires an immediate bolstering of the nation’s system to ensure safe food and laboratory capacity to quickly and accurately identify pathogens, conduct surveillance, and prevent future risks.

In this context, the KAP model is a convenient tool for describing food safety awareness and self‑reported behaviors in different populations [24]. It assumes a sequential pathway in which knowledge shapes attitudes, and attitudes in turn lead to safer, more consistent practices. However, growing evidence shows that this linear cause–effect logic is only partially supported in food safety contexts. Many reviews and empirical studies report that improvements in knowledge or attitudes often do not translate into proportional changes in behavior, with roughly half of food safety training or KAP studies finding no clear alignment between what people know, what they think, and what they actually do in practice [25,26]. Structural equation and mediational models further indicate that the link from knowledge to practice is indirect, culture‑ and context‑dependent, and frequently mediated or moderated by attitudes, perceived control, risk perception, and environmental constraints [27–29]. Overall, the KAP framework tends to underestimate social, cultural and situational influences on behavior and appears better suited for establishing a descriptive baseline and identifying at‑risk groups than for predicting behavior change on its own.

Thus, in the present study, the KAP framework was used as a descriptive and exploratory tool rather than as a presumption of a simple KAP causal pathway. Although KAP measures cannot fully explain why knowledge and attitudes translate into, or fail to translate into, safe food-handling behavior, they remain useful for establishing a baseline, identifying gaps in KAP, and characterizing population groups that may require targeted interventions. Accordingly, the present study uses KAP assessment to characterize food safety KAP among Bangladeshi secondary school students and to examine their variation across relevant sociodemographic and educational characteristics. International KAP studies among consumers, students and food handlers consistently document awareness shortfalls and inconsistent practices, even among respondents with positive attitudes or adequate knowledge [25,26]. This persistent knowledge–practice gap underscores the need to interpret KAP data cautiously and to complement it with behavior‑oriented theories and methods when designing interventions.

Despite the ubiquitous nature of food safety concerns in Bangladesh and the evident susceptibility of the youth, prior research has taken a somewhat limited approach [30–35]. Evidence from Bangladesh shows that adult consumers and household food handlers generally have only moderate knowledge and suboptimal food safety practices [34,35]. National and community-based studies report unsafe behaviors, particularly in handwashing, temperature control, storage, and leftover handling [35,36]. Similarly, studies among household food handlers and university students reveal low understanding of key food safety domains such as food poisoning, cross-contamination, and safe storage, with socio-economic factors such as income, education, and residence influencing KAP levels [30,34]. Research on street food consumers further indicates only moderate awareness of food safety risks, with younger and less-educated groups being the least informed [37]. Comparable findings from other developing countries show that even when awareness exists, unsafe food handling practices remain common [38]. Collectively, this evidence suggests that food safety challenges in Bangladesh extend across households and consumers; however, school-aged adolescents—emerging consumers and future household food handlers—remain under-researched.

Importantly, the study did not rely on KAP measures alone. The Geographic Information System (GIS) component was incorporated to address a distinct limitation of conventional KAP surveys: while KAP analysis can identify which population groups exhibit poorer knowledge or practices, it does not indicate whether these vulnerabilities are geographically concentrated. GIS enables the integration of individual-level attributes with geographic location to visualize spatial variation, identify potential clusters or hotspots, and reveal geographical patterns that may remain obscured in conventional statistical summaries [39,40]. Recent geospatial studies have demonstrated the value of this approach for identifying heterogeneous distributions and localized risk patterns in foodborne and waterborne disease surveillance, as well as for supporting geographically targeted resource allocation and intervention planning [41–43]. Thus, the rationale for combining KAP and GIS in the present study was complementary: KAP analysis was used to identify “who” may be vulnerable and which behavioral domains require attention, whereas GIS was used to examine “where” these vulnerabilities are geographically concentrated. This integrated approach was intended to provide a more actionable evidence base for developing geographically and contextually tailored food safety education and prevention strategies for Bangladeshi school students.

A notable and crucial knowledge gap exists among secondary school students. This group has received little attention in research to date, yet early evidence suggests both poor knowledge and incongruent self-reported behavior [39–41]. In particular, this population is critical, as adolescence constitutes a key transition in gaining autonomy in food choice and intake. They are the highest consumers of high-risk street foods and frequently participate in initial food handling and preparation within the home; however, they are the age group with the least access to hygienic/safe food information, leading them to be more exposed to the risk of foodborne disease. Disregarding this as a high-priority target group, policymakers may squander an important strategic opportunity to mold lifelong safe food-handling behaviors at a point in development when they are most conducive.

The selection of secondary school students in Bangladesh is further justified by several school-based food-related illness incidents reported in the country. Investigation of an acute illness episode among schoolchildren consuming high-energy biscuits in a school feeding program in northwest Bangladesh demonstrated how school-based food incidents can rapidly affect many students [42]. In another recent incident, more than 40 students from Kazirhat High School in Pabna district reportedly fell unconscious during school hours, where food poisoning was considered a possible cause by health authorities [43]. Similarly, in Noakhali district, a madrasa student died and several other students became ill following suspected food poisoning after consuming food provided at their educational institution [44]. These incidents indicate that Bangladeshi school-going adolescents are directly exposed to food safety risks within educational settings, were contaminated food, unhygienic environments, or unsafe food handling practices may rapidly affect a large number of students. Therefore, secondary school students represent an important and vulnerable population for assessing food safety KAP and for designing effective preventive public health interventions.

Despite these growing concerns, we have very limited empirical knowledge on food safety KAP among Bangladeshi school-going children, and there is a lack of research related to their spatial variations across Bangladesh [28–33]. Therefore, the present study was undertaken to determine the food safety KAP level of secondary school students in Bangladesh with the objective of assessing whether any significant effect on KAP could be attributed to selected fundamental domains such as socio-demographic characteristics and mapping geographical variations with a GIS-based approach. The results will inform policymakers, educators, and public health practitioners to develop tailored interventions to facilitate safer food handling practices among adolescents.

## Methodology

### Ethics statement

This study was approved by the Research Ethics Committee of Patuakhali Science and Technology University (Approval NO: PSTU/IEC/2023/57). This study was conducted in compliance with the ethical principles of the Declaration of Helsinki (1975, revised 2008). No financial or academic incentives were provided and participation was voluntary. Prior to data collection, the aim of the study, confidentiality guarantee, and participants’ right to withdraw from the study without consequences at any time were presented to them. All participants provided written consent before completing the questionnaire. Data confidentiality was strictly maintained and anonymized before analysis was conducted.

### Study design and setting

A cross-sectional descriptive design was used in this study to determine KAPs towards food safety hazards among Bangladeshi secondary school students. This study covered eight districts, one for each of the eight divisions in Bangladesh: Dhaka, Chattogram, Rajshahi, Khulna, Sylhet, Barishal, Mymensingh, and Rangpur. Six secondary schools were selected from each district (three from urban areas and three from rural areas). The inclusion of districts from all divisions yielded a diverse sample that would reflect differences in socio-economic status, educational facilities and infrastructure, cultural behaviors, and environmental circumstances within the secondary school student population in Bangladesh, thus strengthening the external validity of the results to the general secondary school student population.

### Inclusion and exclusion criteria

The eligibility criteria were as follows: (i) aged between 12 and 17 years; (ii) currently studying in class nine or ten, which represent the secondary education level in Bangladesh; (iii) able to read and understand Bangla; and (iv) information provided about the objectives of this research project with assurance that it is totally voluntary, as well as given to obtain written consent for participating in the study. The age range of 12–17 years reflects natural variation in school entry age, grade repetition, and academic progression within the Bangladeshi education system. Exclusion criteria were also applied to student-level data (e.g., students not present on the day of the survey, did not meet the age or grade criteria). Thus, these criteria ensured that adolescents had adequate cognitive function to understand and respond accurately to the questionnaire items.

### Sampling technique and sample size calculation

**Fig 1:**
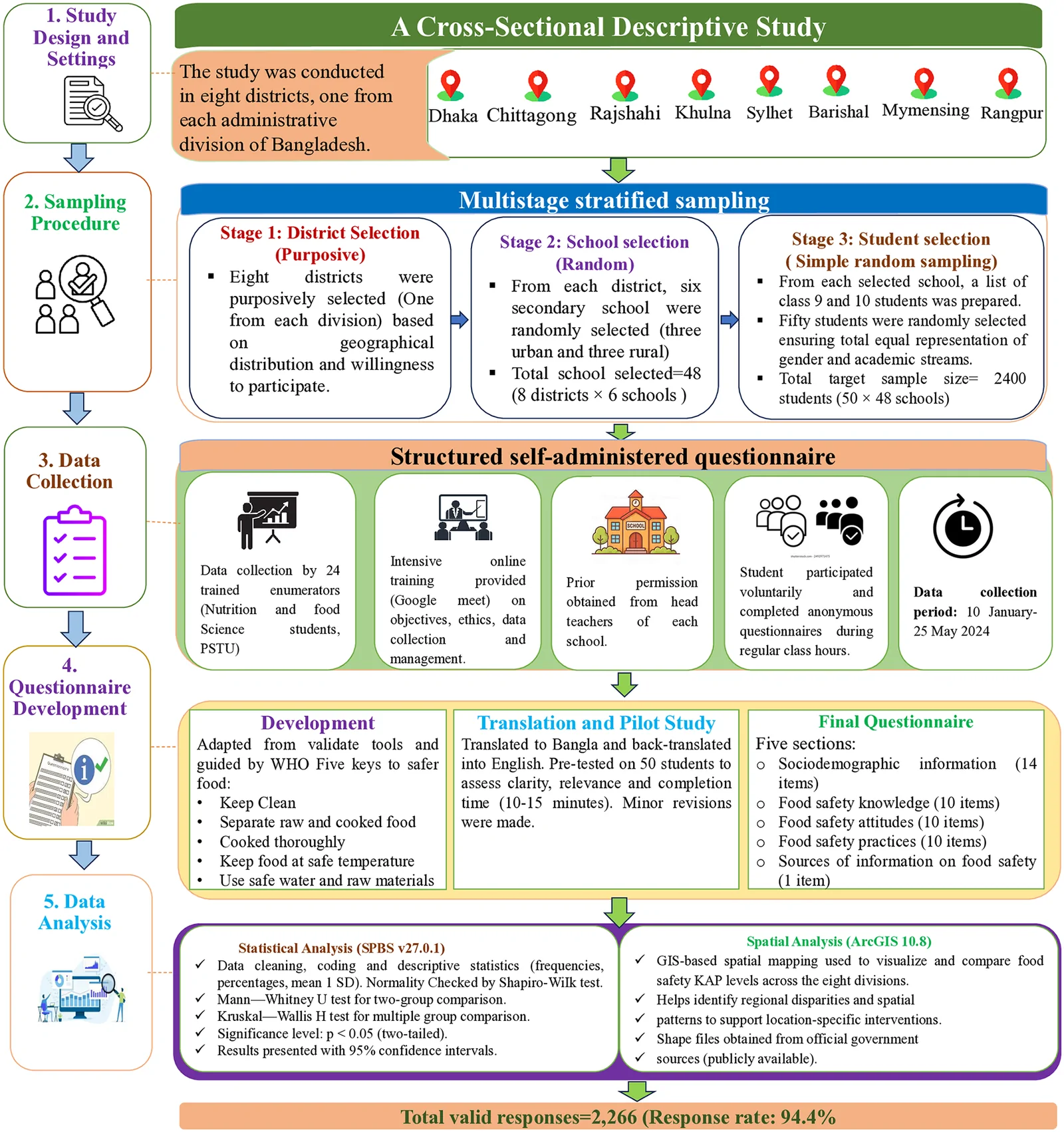
Flowchart of the research methodology.

The survey used a multistage stratified sampling. In the first phase, eight districts (one per each administrative division) were purposively chosen to ensure broad geographic coverage across the country and willingness of schools to participate. Although this stage involved purposive selection, inclusion across all administrative divisions was intended to enhance national-level representativeness of secondary school settings. These were treated as eight separate strata. In the second stage, three urban and three rural secondary schools were randomly selected from each district based on the type of school and location, to ensure representation of both urban and rural educational environments and reduce location-based selection bias, resulting in a total of 48 schools. Importantly, school location (urban/rural) was used only as a sampling criterion at the school selection stage, whereas participants’ residential area (urban/rural) was collected separately as an individual-level sociodemographic variable and was not restricted by school location. In the last stage, stratified random sampling was performed within each school using the list of enrolled students in grades 9 and 10. Fifty students were selected randomly to ensure equal proportions of males, females, and academic streams, thereby minimizing selection bias at the participant level. This multi-stage process culminated in a desired target sample of 2400 students (50 × 48 schools). A flowchart summarizing the study methodology and participant selection process is presented in Fig 1.

The initial required sample size was calculated for a simple random sample using Cochran’s formula [45] for cross-sectional studies:


$$\begin{array}{c} n=\frac{Z^{\text{2}\;}\;.\;p\;.\;(\text{1}-p)}{d^{\text{2}\;}} \end{array}$$

Where, Z = 1.96 (for 95% confidence level), *p* = 0.5 (assumed proportion of adequate knowledge to maximize sample size), and d = 0.03 (margin of error).


$$\begin{array}{c} n=\frac{{\left(\text{1}.\text{9}\text{6}\right)}^{\text{2}\;}\;\times \text{0}.\text{5}\;\times \;(\text{1}-\text{0}.\text{5})}{{\left(\text{0}.\text{0}\text{3}\right)}^{\text{2}\;}}\;=\;\frac{\text{3}.\text{846 }\times \text{0}.\text{2}\text{5}}{\text{0}.\text{0009}}\;\approx \text{1}\text{0}\text{6}\text{7} \end{array}$$

Because the actual design was multistage stratified cluster sampling (districts → schools → students), this simple random sample-based sample size was then inflated by a design effect to account for clustering and stratification. Assuming a design effect (DEFF) of 2.0, a commonly used conservative value in school-based multistage surveys, the target sample size became:


$$\begin{array}{c} n=n_{0}\times DEFF=1,067\times 2\approx 2,134 \end{array}$$

To further ensure adequate numbers within each stratum and allow for potential non‑response, this figure was rounded up to a planned sample of 2,400 students (50 students in each of 48 schools). In practice, 2,226 valid questionnaires were obtained, corresponding to a 94.4% response rate, which is sufficient to maintain the planned precision for prevalence estimates under the chosen design effect.

### Data collection procedure

A structured, self-administered questionnaire was distributed in classrooms from 10 January to 25 May 2024 to collect data. Twenty-four trained enumerators the final year undergraduate students of the Nutrition and Food Science Department of Patuakhali Science and Technology University (PSTU) were formed into the data collection group. All enumerators received an intensive (online) training workshop (using Google Meet), which was held prior to data collection and focused on study objectives, ethical issues, participant engagement, data management, and techniques to avoid interviewer bias. The division uses three enumerators per locality to consolidate an equal application and monitor the process. Prior consent was obtained from the head teacher at each school. Following a description of the objectives and methods of the study, the students were asked to participate voluntarily. Under the supervision of trained data collectors, students filled out anonymous questionnaires during regular class hours, with class teachers available to offer assistance and provide a supportive environment that would enhance accurate responses during the questionnaire completion.

### Questionnaire development and pilot testing

A structured questionnaire was designed to measure the food safety KAP among Bangladeshi secondary students. To support construct validity, the questionnaire was adapted from a combination of pre-validated questionnaires developed by Tutu [46], Cheng [47], and Kuo and Weng [48]. The items were culturally adapted to reflect Bangladeshi-specific food handling, storage, and consumption behaviors. In addition, the questionnaire development was guided by the World Health Organization (WHO) Five Keys to Safer Food principles, including keeping clean, separating raw and cooked food, cooking food thoroughly, keeping food at safe temperatures, and using safe water and raw materials. These principles were incorporated into the development of several knowledge and practice items related to food hygiene and safe food handling behaviors, thereby ensuring theoretical and content relevance of the instrument. The semantic and conceptual equivalence of the Bangla version of the questionnaire was checked after translation and back-translation into English by two bilingual translators. A pre-test was also conducted among 50 students to test question clarity, relevance, consistency of response, and completion time. After pilot testing, minor changes in wording and context were made to improve readability and cultural relevance. The results from the pilot phase were not included in the final analysis. The mean time to completion was approximately 10–15 min. The final questionnaire consisted of 5 sections.

1) Sociodemographic information (14 items)
2) Food safety knowledge (10 items)
3) Food safety attitudes (10 items)
4) Food safety practices (10 items)
5) Sources of information on food safety (1 item)

### Measures/variables

#### Sociodemographic information.

Sociodemographic information was collected using a structured questionnaire and included participants’ age and gender, current academic class (9 or 10) and study group (Science, Humanities, or Business), and type of school attended (government, half government/MPO, or private). Data were also obtained on parents’ highest level of education and occupation, permanent residential area (urban or rural), and family monthly income (<15,000 BDT, 15,000–30,000 BDT, or >30,000 BDT) (122.37 BDT = 1 USD). Additionally, information on household responsibilities was recorded, including the primary person responsible for grocery shopping and participants’ involvement in food preparation (daily, sometimes, once a week, once a month, or never), along with previous experience of food poisoning. The explanatory variables included in this study were selected a priori based on their theoretical relevance and prior food safety KAP literature [30–41], as they plausibly influence students’ access to food safety information, opportunities for practical learning, and day-to-day food-handling behavior.

### Food safety knowledge assessment

Food safety knowledge was measured using a 10-items tool that was based on validated tools published elsewhere and tailored for Bangladeshi culture. The items covered key aspects of handling, storage, hygiene, and safety. Correct answers were scored as 1, and incorrect or “I don’t know” responses were scored as 0. Three of the items (K2, K3, and K7) had negative wording and were reverse-scored to maintain the scoring direction. For example, one item read, “People should not cook food when they are diseased or ill.” The knowledge scores ranged from 0 to 10 points, with a higher number of points indicating more knowledge. The knowledge items demonstrated good internal consistency, based on Cronbach’s alpha, which was 0.712.

### Food safety attitudes assessment

A 10-item questionnaire derived from validated scales and tailored to the context of Bangladesh was used to assess participants’ perceptions of food safety. Each response was scored on a scale of 1 (strongly disagree) to 5 (strongly agree), and the sum of scores for each item yielded higher scores, indicating more positive attitudes toward safe food handling and consumption. Reverse coding was assigned to negatively worded items (A1, A2, and A4) to standardize scoring. For instance, one of the items read “It is important for students to receive training as a measure to reduce food contamination risks.” The total attitude scores ranged from 10 to 50, with higher scores representing more positive attitudes toward food safety practices. The attitude items were internally consistent (Cronbach’s alpha = 0.704).

### Food safety practices assessment

Food safety practices (as reported by the participants) were measured using a 10-item scale used in previous studies and adapted for context-specific relevance to Bangladesh. Each item was scored according to the level of agreement on a five-point Likert scale from 1 (never) to 5 (almost always), where higher scores indicated a greater frequency of practicing safe food-handling behaviors. Negatively stated items (P2 and P3) were inverse-coded for the ease of scoring. For example, one participant asked, “I store raw sausage (sausage meat without a wrapper) with other foods in the fridge.” The total practice score ranges from 10 to 50 points. The internal consistency of the practice items was acceptable, with a Cronbach’s alpha value of 0.758.

### Statistical analysis

Data were analyzed using IBM SPSS Statistics (version 27.0.1; IBM Corp., Armonk, NY, USA). Data cleaning and coding were performed before the analysis. Descriptive statistics, including frequencies, percentages, means, and standard deviations, were calculated to summarize sociodemographic characteristics and KAP distributions. As the KAP scores were not normally distributed (confirmed by Shapiro–Wilk tests), non-parametric tests were applied. The Mann–Whitney U test was used to compare the median KAP scores between the two groups, while the Kruskal–Wallis H test was used for comparisons across multiple groups.

In addition to conventional statistical analyses, GIS mapping using ArcGIS 10.8 was employed as a complementary analytical approach to visualize and explore spatial heterogeneity in food safety KAP across the eight administrative divisions of Bangladesh. The primary purpose of incorporating GIS was not to perform inferential spatial modeling, but to enhance interpretability by identifying geographical patterns, regional clustering, and disparities that may remain hidden in aggregated statistical outputs. This spatial representation provides an added analytical dimension by linking observed variations in KAP outcomes to contextual differences across regions. The maps were created using shape files received from official government mapping websites that are freely available to the public. The GIS outputs were used to visually compare regional differences and support policy-relevant interpretation of findings, particularly for identifying priority areas for targeted food safety education and intervention programs. All tests were two-tailed, and a p < 0.05 significance level was considered statistically significant. The results are presented with 95% confidence intervals, where appropriate.

## Results

### Sociodemographic profile of respondents

**Table 1** displayed sociodemographic characteristics of the participants’ sociodemographic characteristics. The mean age was 15.26 years (SD = 1.02). More than half of the participants were boys (51.1%, n = 1,137), the majority were aged 15–17 years (75.6%, n = 1,682), and enrolled in class 9 (50.4%, n = 1,122). Most students were from the science stream (63.7%, n = 1,417) and had attended half-government/MPO schools (57.3%, n = 1,276). Regarding parental education, 28.9% (n = 644) of fathers and 38.2% (n = 851) of mothers completed secondary education. The majority of the students resided in rural areas (69.2%, n = 1540) and came from families with a monthly income between 15,000 and 30,000 BDT (52.8%, n = 1,176). Nearly half (48.1%, n = 1,070) reported occasional involvement in food preparation, and (40.1%, n = 893) had experienced foodborne illnesses.

**Table 1:** Sociodemographic characteristics of the study participants.

Variables	Categories	Frequency	Percentage %
**Age of the participant** [15.26 ± 1.02]			
	12-14 years	544	24.4%
	15-17 years	1682	75.6%
**Gender of the participant**			
	Boy	1137	51.1%
	Girls	1089	48.9%
**Current academic class**			
	Class-9	1122	50.4%
	Class-10	1104	49.6%
**Study group**			
	Science group	1417	63.7%
	Humanities group	627	28.1%
	Business group	182	8.2%
**Studying school type**			
	Government school	713	32.1%
	Half government/MPO school	1276	57.3%
	Private school	237	10.6%
**Father’s education**			
	No formal education	194	8.7%
	Primary	331	14.9%
	Secondary	644	28.9%
	Higher secondary	550	24.7%
	Hons and above	507	22.8%
**Mother’s education**			
	No formal education	183	8.2%
	Primary	396	17.8%
	Secondary	851	38.2%
	Higher secondary	503	22.6%
	Hons and above	293	13.2%
**Permanent resident**			
	Rural area	1540	69.2%
	Urban area	686	30.8%
**Family monthly income**			
	<15000 BDT	473	21.3%
	15000-30000 BDT	1176	52.8%
	> 30000 BDT	577	25.9%
**Father’s occupation**			
	Job holder	768	34.5%
	Farmer/Fisherman/Rickshaw puller/ labor	625	28.1%
	Business/self-dependent	643	28.9%
	Other’s^a^	190	8.5%
**Mother’s occupation**			
	Job holder	226	10.1%
	Housewife	1883	84.6%
	Business	46	2.1%
	Other’s^b^	71	3.2%
**The person responsible for buying groceries**			
	Father	1810	81.3%
	Mother	307	13.8%
	Other’s^c^	109	4.9%
**Involvement in food preparation**			
	Yes, daily	665	29.9%
	No, never	248	11.1%
	Yes, once a week	178	8.0%
	Yes, once a month	65	2.9%
	Sometimes	1070	48.1%
**Experience of food poisoning**			
	Yes	893	40.1%
	No	1333	59.9%

Note: Abbreviations: BDT, Bangladeshi Taka; 122.37 BDT = 1 USD.

a retired or pensioner, Driver, Unemployed,

b Day labour, tailor, Small trader/ Shopkeeper, entrepreneur

c Older siblings, grandfather, uncle, caretakers

### Sources of knowledge and food choices based on the environment

**Fig 2:**
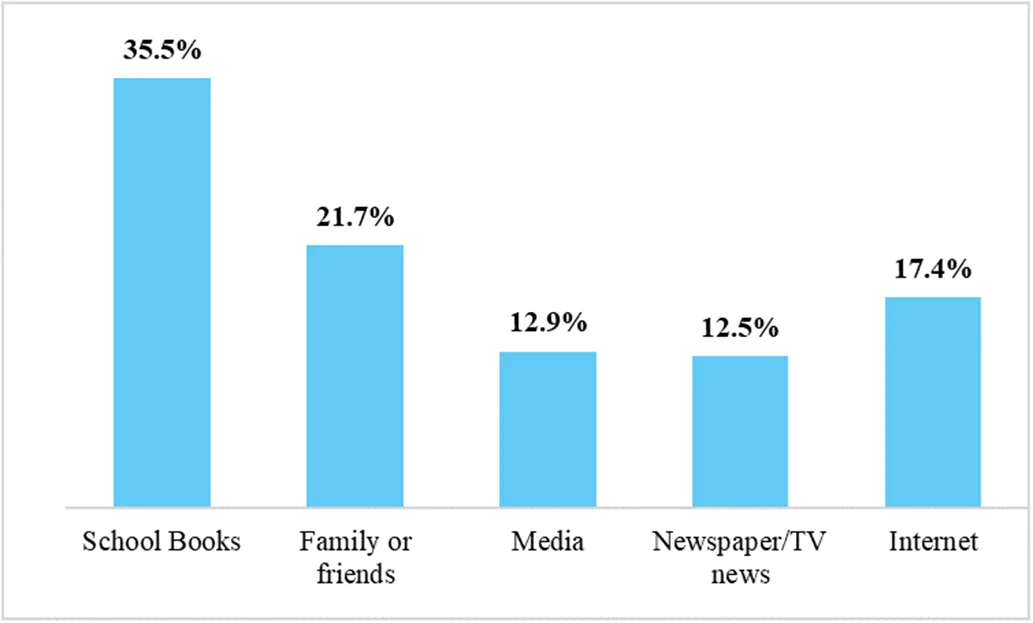
Sources of information on food safety reported by participants.

The majority of the students gathered food safety knowledge from academic books (35.5%), followed by 21.7% from family or friends, and 17.4% from the internet (Fig 2).

### Food safety knowledge

The overall correct responses were 50.4% (5.04/10 × 100), and the mean knowledge score was 5.04 on ten scales (SD = 2.53). The majority of students recognized the need for hand washing before eating (68.7%, K9) and for not preparing food while unwell (67.2%, K1) (**Table 2**). Moderate knowledge was achieved for safekeeping raw meat (51.7%, K2) and ready-to-eat foodstuffs (53.4%, K3), and handling leftovers appropriately (K4-6, 42.2–48.8%). The lowest level of knowledge was related to spoiled foods (19.7%; K7). Furthermore, 66.9% knew that it was important to wash fruit and vegetables (K10). Overall, students showed satisfactory knowledge of personal hygiene, and ignorance was found in food storage, leftover management, and expired foods. Higher knowledge scores were observed among younger students (12–14 years), Class 9 students, those in the science group, students whose parents had higher educational attainment, and those from higher-income families. Knowledge scores also varied according to fathers’ occupation and level of involvement in food preparation **(Table 5)**.

**Table 2:** Knowledge of the study participants regarding food safety.

SL	Statement	Yes (%) ^a^
K1	People should not prepare food when they suffer from a disease or illness.	1496 (67.2)
K2	The ideal place to store raw meat in the refrigerator is on the upper shelf. ^Ϯ^	1151 (51.7)
K3	The ideal place to store ready-to-eat foods like cake and bread is on the bottom shelf. ^Ϯ^	1188 (53.4)
K4	The best way to handle leftover food is to put it in the refrigerator within 2 hours of cooking it.	1087 (48.8)
K5	If leftover food looks and/or smells good, it is still not safe to eat.	973 (43.7)
K6	Reheating leftover food kills all germs in it.	939 (42.2)
K7	Expired foods are safe to eat. ^Ϯ^	493 (19.7)
K8	The best way to refrigerate large quantities of hot food is to divide them into small portions and store them in shallow/wide containers.	938 (42.1)
K9	Hand washing with soap and water before eating food reduces the chance of food poisoning.	1530 (68.7)
K10	Rinsing fruits and vegetables under running tap water/tube well thoroughly can decrease the chance of food poisoning.	1489 (66.9)

Note: ^a^ correct response frequency and percentage

ϮItems are reversely coded.

SD: Standard deviation

### Food safety attitudes

In terms of food safety attitude, the overall correct answer rate was 49.1% (24.56/50 × 100), indicating a borderline positive attitude. The mean total score was 24.56 points (SD = 6.80, range: 10–50) out of a possible 50.0 points. Participants’ attitudes towards food safety differed in various dimensions (Table 3). Some students had positive attitudes toward hygiene and risk prevention, the majority of whom agreed with the importance of food hygiene training (A7, 54.8% strongly disagreed) and food that had a bad smell, appearance, or taste was unfit-to-eat (A9, 49.6% strongly disagreed). Similarly, 54.2% strongly disagreed that it was not necessary to wash their hands before handling food (A10). However, some items were misunderstood: a large percentage of students thought it was safe to eat food additives (A1, 36.1% strongly not safe) and that expired foods have a good appearance and can be eaten (A2, 37.5% strongly agree). Higher attitude scores were observed among boys, Class 9 students, science-group students, those attending government schools, and students who reported preparing food once a week. (Table 5).

**Table 3:** Attitude of the study participants regarding food safety.

SL	Statement	Mean ± SD	Strongly agree	Agree	Undecided	Disagree	Strongly Disagree
A1	I think that those food products containing additives are safe. ^Ϯ^	3.62 ± 1.423	333 (15.0%)	186 (8.4%)	283 (12.7%)	620 (27.8%)	804 (36.1%)
A2	I think those expired food products with good appearance and no odor can still be eaten. ^Ϯ^	2.35 ± 1.377	835 (37.5%)	575 (25.8%)	223 (10.0%)	380 (17.1%)	213 (9.6%)
A3	I think that food products preserved for a long time in supermarkets must have preservatives.	2.14 ± 1.174	143 (6.4%)	155 (7.0%)	394 (17.7%)	721 (32.4%)	813 (36.5%)
A4	I think that opening canned food products before their expiration dates do not spoil them. ^Ϯ^	3.11 ± 1.416	433 (19.5%)	610 (27.4%)	405 (18.2%)	322 (14.4%)	456 (20.5%)
A5	I think that a good part of a rotten fruit can still be eaten after the moldy part is removed.	2.60 ± 1.349	270 (12.1%)	386 (17.3%)	310 (13.9%)	700 (31.4%)	560 (25.2%)
A6	I think flavoring agents, such as sugar and salt, enhance food flavor, so those additives have no harmful effects on human health.	2.96 ± 1.456	415 (18.6%)	532 (23.9%)	368 (16.5%)	371 (16.7%)	540 (24.3%)
A7	Food hygiene training for students is an important issue in reducing the risk of food contamination.	1.76 ± 1.086	112 (5.0%)	92 (4.1%)	160 (7.2%)	642 (28.8%)	1220 (54.8%)
A8	You are concerned about the safety of food in restaurants around the school.	2.18 ± 1.207	151 (6.8%)	197 (8.8%)	353 (15.9%)	715 (32.1%)	810 (36.4%)
A9	Food with a bad smell, appearance, or taste is unsuitable for consumption.	2.01 ± 1.298	196 (8.8%)	173 (7.8%)	187 (8.4%)	565 (25.4%)	1105 (49.6%)
A10	Hand washing before handling and eating food reduces the risk of contamination.	1.84 ± 1.195	156 (7.0%)	117 (5.3%)	153 (6.9%)	593 (26.6%)	1207 (54.2%)

Note: ^Ϯ^ Items are reversely coded.

SD: Standard deviation

### Food safety practices

The mean practice score was 19.92 out of 50 (SD = 6.78), corresponding to a correct practice rate of 39.8% (19.92/50 × 100), indicating generally poor compliance with recommended food safety practices. Participants’ self-reported food safety practices revealed suboptimal adherence to recommended practices (Table 4). Less than one-third reported consistently reheating leftover food before eating (P1, 20.0% combining “almost always” and “often”). Additionally, 34.0% reported that they would consume leftover food without reheating if it appeared or smelled acceptable (P2), indicating reliance on sensory cues when judging food safety. Unsafe storage practices were common, with 44.0% keeping raw and cooked food together in the fridge (P3) and 46.6% not storing leftovers in cool places (P4). Preventive practices were also limited: only 3.6%–4.2% consistently checked expiry dates (P5) or discarded expired foods (P7) and 10.1% avoided foods with unknown additives (P6). Proper hand and food hygiene were infrequent, with 62.8%–67.3% rarely washing hands before eating or after outdoor activities (P9–P10) and 67.2% seldom rinsed fruits thoroughly (P8). Higher practice scores were observed among boys, students attending government schools, those whose parents had no formal education, students whose fathers were farmers, fishermen, rickshaw pullers, or laborers, and those involved in food preparation once a week. Students who reported previous food poisoning experience also demonstrated higher practice scores than those without such experience (Table 5).

**Table 4:** Practice of the study participants regarding food safety.

SL	Statement	Mean ± SD	Almost Always	Often	Sometimes	Seldom	Never
P1	Before eating, reheat leftover food.	2.45 ± 1.296	208 (9.3%)	238 (10.7%)	631 (28.3%)	418 (18.8%)	731 (32.8%)
P2	I will eat leftover food without reheating, if it looks and/or smells good. ^Ϯ^	2.58 ± 1.431	291 (13.1%)	379 (17.0%)	426 (19.1%)	374 (16.8%)	756 (34.0%)
P3	I keep raw food and cooked food in the same place in the fridge. ^Ϯ^	2.40 ± 1.499	333 (15.0%)	257 (11.5%)	346 (15.5%)	311 (14.0%)	979 (44.0%)
P4	I keep my leftover food in the fridge/cool places.	1.94 ± 1.122	102 (4.6%)	130 (5.8%)	335 (15.0%)	621 (27.9%)	1038 (46.6%)
P5	I check the expiry date of the product before buying.	1.66 ± 1.070	80 (3.6%)	107 (4.8%)	225 (10.1%)	384 (17.3%)	1430 (64.2%)
P6	I avoid eating foods that contain unknown additives.	2.38 ± 1.336	224 (10.1%)	263 (11.8%)	436 (19.6%)	516 (23.2%)	787 (35.4%)
P7	Throw away foods that have passed the expiration date.	1.63 ± 1.095	93 (4.2%)	111 (5.0%)	184 (8.3%)	329 (14.8%)	1509 (67.8%)
P8	Thoroughly wash/rinse fruits under running water before eating.	1.60 ± 1.025	72 (3.2%)	86 (3.9%)	212 (9.5%)	360 (16.2%)	1496 (67.2%)
P9	I wash my hands with soap and under running water when I get home from school or after playing outside.	1.67 ± 1.052	73 (3.3%)	107 (4.8%)	225 (10.1%)	424 (19.0%)	1397 (62.8%)
P10	I wash my hands with soap and under running water before eating food.	1.61 ± 1.052	84 (3.8%)	83 (3.7%)	213 (9.6%)	347 (15.6%)	1499 (67.3%)

Note: ^Ϯ^ Items are reversely coded.

SD: Standard deviation

**Table 5:** Association between socio-demographic characteristics and food safety knowledge, attitude, and practice scores.

Variables	Category	Knowledge (Mean ± SD)	*U/H*	*p-*value	Effect Size r/ε²	Attitude (Mean ± SD)	*U/H*	*p-value*	Effect Size r/ε²	Practice (Mean ± SD)	*U/H*	*p-value*	*Effect Size* *r/ε²*
**Age of the participant** [15.26 ± 1.019]													
	12-14 years	5.38 ± 2.528	413444.00	**<0.001** ^**a**^	0.072	25.18 ± 7.160	435737.00	0.094^a^	0.035	20.18 ± 7.685	453748.00	0.773^a^	0.006
	15-17 years	4.94 ± 2.521				24.36 ± 6.678				19.83 ± 6.473			
**Gender of the participant**													
	Boy	5.17 ± 2.422	594246.50	0.098^a^	0.035	25.10 ± 6.599	550051.00	<0.001^a^	0.097	21.24 ± 6.959	459795.00	<0.001^a^	0.223
	Girls	4.91 ± 2.632				24.01 ± 6.976				18.53 ± 6.319			
**Current academic class**													
	Class-9	5.33 ± 2.519	539866.50	<0.001^a^	0.112	25.28 ± 7.233	556057.50	<0.001^a^	0.089	19.94 ± 7.252	601081.50	0.228^a^	0.026
	Class-10	4.76 ± 2.509				23.83 ± 6.262				19.90 ± 6.288			
**Study group**													
	Science group	5.25 ± 2.453	23.87	<0.001	0.010	24.89 ± 6.798	8.98	0.011	0.003	20.18 ± 6.801	8.36	0.015	0.003
	Humanities group	4.62 ± 2.680				23.96 ± 6.927				19.47 ± 6.978			
	Business group	4.93 ± 2.410				24.08 ± 6.299				19.37 ± 5.895			
**Studying school type**													
	Government	5.06 ± 2.528	0.83	0.660	0.000	25.06 ± 6.947	10.36	0.006	0.004	21.63 ± 7.252	72.74	<0.001	0.032
	Half government/MPO school	5.01 ± 2.557				24.33 ± 6.741				19.21 ± 6.611			
	Private	5.20 ± 2.388				24.35 ± 6.679				18.59 ± 5.133			
**Father’s education**													
	No formal education	4.70 ± 2.523	20.13	<0.001	0.007	25.14 ± 6.526	8.72	0.068	0.002	21.36 ± 6.923	25.23	<0.001	0.010
	Primary	4.78 ± 2.668				23.59 ± 6.517				19.09 ± 6.354			
	Secondary	4.87 ± 2.436				24.13 ± 5.998				19.34 ± 5.913			
	Higher secondary	5.21 ± 2.411				24.56 ± 6.518				19.59 ± 6.624			
	Hons and above	5.39 ± 2.635				25.54 ± 8.124				20.98 ± 7.946			
**Mother’s Education**													
	No formal education	4.59 ± 2.439	19.46	<0.001	0.007	25.12 ± 6.741	8.21	0.084	0.002	22.27 ± 7.040	38.95	<0.001	0.016
	Primary	4.91 ± 2.605				24.13 ± 6.323				19.56 ± 6.240			
	Secondary	5.03 ± 2.356				24.06 ± 5.808				19.24 ± 5.629			
	Higher secondary	5.05 ± 2.690				24.70 ± 7.753				19.77 ± 7.662			
	Hons and above	5.54 ± 2.622				26.05 ± 8.107				21.14 ± 8.246			
**Permanent resident**													
	Rural	5.07 ± 2.479	523081.00	0.771^a^	0.008	24.58 ± 6.633	523383.00	0.729^a^	0.007	19.96 ± 6.734	518209.00	0.474^a^	0.015
	Urban	4.98 ± 2.640				24.54 ± 7.184				19.83 ± 6.915			
**Family monthly income**													
	<15000 BDT	4.53 ± 2.804	39.40	<0.001	0.017	23.99 ± 7.444	5.03	0.081	0.001	19.81 ± 7.317	0.42	0.811	0.000
	15000-30000 BDT	4.99 ± 2.437				24.39 ± 6.426				19.91 ± 6.503			
	>30000 BDT	5.58 ± 2.379				25.39 ± 6.948				20.02 ± 6.920			
**Father’s Occupation**													
	Job holder	5.13 ± 2.615	8.05	0.045	0.002	24.51 ± 7.345	2.17	0.538	0.000	19.76 ± 6.943	22.76	<0.001	0.009
	Farmer/Fisherman/Rickshaw puller/Labor	4.82 ± 2.528				24.58 ± 6.585				20.74 ± 6.934			
	Business/self-dependent	5.16 ± 2.429				24.67 ± 6.364				19.61 ± 6.425			
	Others	5.05 ± 2.488				24.35 ± 6.756				18.88 ± 6.655			
**Mother’s Occupation**													
	Job holder	5.21 ± 2.691	3.80	0.284	0.000	24.92 ± 8.002	1.97	0.579	0.000	20.96 ± 7.517	5.11	0.164	0.001
	Housewife	5.02 ± 2.499				24.49 ± 6.596				19.74 ± 6.636			
	Business	5.57 ± 2.177				24.61 ± 5.235				20.13 ± 5.753			
	Others	4.86 ± 2.968				25.48 ± 8.839				21.03 ± 8.521			
**The person responsible for buying groceries**													
	Father	5.04 ± 2.548	0.16	0.924	0.000	24.47 ± 6.808	3.92	0.141	0.001	19.85 ± 6.830	0.94	0.625	0.000
	Mother	5.04 ± 2.424				25.05 ± 6.836				20.07 ± 6.299			
	Others	5.11 ± 2.540				24.69 ± 6.717				20.58 ± 7.433			
**Involvement in food preparation**													
	Yes, daily	4.85 ± 2.500	35.23	<0.001	0.014	24.42 ± 6.528	23.87	<0.001	0.009	19.79 ± 6.552	34.03	<0.001	0.014
	No, never	4.42 ± 2.610				23.33 ± 6.854				20.90 ± 6.841			
	Yes, once a week	4.99 ± 2.265				25.94 ± 5.877				21.24 ± 5.893			
	Yes, once a month	5.00 ± 2.437				25.38 ± 6.552				20.95 ± 7.129			
	Sometimes	5.32 ± 2.543				24.66 ± 7.077				19.48 ± 6.992			
**Experience of food poisoning**													
	Yes	5.14 ± 2.590	573554.00	0.142 ^a^	0.031	24.81 ± 7.160	581241.00	0.374 ^a^	0.020	20.55 ± 7.064	540344.50	<0.001^a^	0.078
	No	4.98 ± 2.487	413444.00			24.40 ± 6.557				19.49 ± 6.567			
**Total**		**5.04 ± 2.530**				**24.56 ± 6.806**				**19.92 ± 6.789**			

Note: ^a^ Mann-Whitney U test was performed; otherwise, Kruskal-Wallis H test was performed. SD: Standard deviation. Effect’s size was calculated for the Kruskal-Wallis H test using the formula: $\varepsilon^{\text{2}}=\frac{H-k+\text{1}}{n-k}$ and for a Mann–Whitney U test, using the formula: $r=\frac{|Z|}{\sqrt{N}}$. p < 0.05 was considered statistically significant. U = Mann–Whitney U statistic; H = Kruskal–Wallis H statistic; BDT = Bangladeshi Taka.

### Factors associated with overall food safety KAP scores

Table 6 summarizes the multiple linear regression results. Overall, the patterns observed in the descriptive findings were partly supported and refined by the regression analysis. The model for food safety knowledge explained a relatively small percentage of the variance (Adjusted R^2^ = 0.057). Several demographic factors were significantly associated with the knowledge scores. Specifically, boys (β = 0.062, *p* = 0.004), students residing in rural areas (β = 0.089, *p* < 0.001), and class 9 students (β = 0.101, *p* < 0.001) exhibited better food safety knowledge. Conversely, students from lower family income groups (both <15,000 BDT and 15,000–30,000 BDT compared with >30,000 BDT) showed significantly lower knowledge scores. Furthermore, having a father with a primary or secondary education level was negatively associated with knowledge. Interestingly, daily involvement in food preparation and never being involved (compared to sometimes) were negatively associated with knowledge scores.

**Table 6:** Relationship between socio-demographic characteristics and food safety knowledge, attitude, and practice among schoolchildren.

Variables	Knowledge ^a^				Attitude ^b^				Practice ^c^			
	β	95% CI		*p-value*	β	95% CI		*p-value*	β	95% CI		*p-value*
		LL	UL			LL	UL			LL	UL	
(Constant)		3.958	5.832	<0.001		14.894	19.240	<0.001		5.264	10.057	<0.001
**Age of the participant**												
12-14 years	0.022	-0.139	0.399	0.345	-0.011	-0.782	0.438	0.580	0.034	-0.108	1.170	0.103
15-17 years	Reference				Reference				Reference			
**Gender of the participant**												
Boy	0.062	0.099	0.531	**0.004**	0.049	0.181	1.162	**0.007**	0.134	1.302	2.331	**<0.001**
Girls	Reference				Reference				Reference			
**Current academic class**												
Class-9	0.101	0.276	0.743	**<0.001**	0.065	0.349	1.412	**0.001**	-0.022	-0.855	0.260	0.296
Class-10	Reference				Reference				Reference			
**Study group**												
Science group	0.051	-0.122	0.658	0.178	0.035	-0.395	1.374	0.278	0.044	-0.311	1.543	0.193
Humanities group	-0.040	-0.642	0.194	0.294	0.039	-0.364	1.531	0.227	0.038	-0.417	1.569	0.255
Business group	Reference				Reference				Reference			
**Studying school type**												
Government school	0.006	-0.343	0.403	0.874	0.049	-0.126	1.563	0.095	0.160	1.442	3.212	**<0.001**
Half government/MPO school	0.007	-0.327	0.396	0.852	0.025	-0.477	1.159	0.414	0.050	-0.175	1.540	0.119
Private school	Reference				Reference				Reference			
**Permanent resident**												
Rural area	0.089	0.231	0.746	**<0.001**	-0.002	-0.610	0.561	0.934	0.022	-0.292	0.935	0.304
Urban area	Reference				Reference				Reference			
**Father’s education**												
No formal education	-0.056	-1.048	0.045	0.072	-0.009	-1.453	1.024	0.734	-0.066	-2.897	-0.302	**0.016**
Primary	-0.077	-1.028	-0.068	**0.025**	-0.072	-2.463	-0.285	**0.013**	-0.118	-3.390	-1.105	**<0.001**
Secondary	-0.082	-0.865	-0.053	**0.027**	-0.041	-1.537	0.305	0.190	-0.109	-2.604	-0.672	**0.001**
Higher secondary	-0.022	-0.490	0.230	0.479	-0.045	-1.530	0.100	0.086	-0.084	-2.174	-0.464	**0.003**
Hons and above	Reference				Reference				Reference			
**Mother’s education**												
No formal education	-0.036	-0.917	0.251	0.264	0.005	-1.209	1.437	0.866	0.068	0.306	3.078	**0.017**
Primary	-0.015	-0.602	0.399	0.691	-0.052	-2.052	0.214	0.112	0.014	-0.946	1.430	0.690
Secondary	-0.030	-0.599	0.290	0.495	-0.079	-2.118	-0.102	**0.031**	0.019	-0.791	1.323	0.622
Higher secondary	-0.066	-0.802	0.003	0.052	-0.030	-1.403	0.421	0.291	0.009	-0.804	1.108	0.755
Hons and above	Reference				Reference				Reference			
**Father’s occupation**												
Job holder	-0.005	-0.437	0.383	0.898	-0.020	-1.216	0.642	0.545	0.005	-0.905	1.041	0.891
Farmer/Fisherman/Rickshaw puller/ labor	0.015	-0.351	0.518	0.706	0.050	-0.229	1.738	0.133	0.109	0.621	2.683	**0.002**
Business/self-dependent	0.024	-0.273	0.545	0.513	0.017	-0.673	1.180	0.592	0.030	-0.522	1.421	0.364
Other’s	Reference				Reference				Reference			
**Mother’s occupation**												
Job holder	0.030	-0.408	0.917	0.452	-0.051	-2.643	0.360	0.136	0.001	-1.551	1.597	0.977
Housewife	0.049	-0.252	0.940	0.258	-0.047	-2.237	0.465	0.199	-0.045	-2.266	0.567	0.240
Business	0.052	0.009	1.856	**0.048**	-0.044	-4.210	-0.020	**0.048**	-0.010	-2.667	1.727	0.675
Other’s	Reference				Reference				Reference			
**Family monthly income**												
<15000 BDT	-0.158	-1.326	-0.629	**<0.001**	0.025	-0.374	1.215	0.299	0.026	-0.403	1.262	0.311
15000-30000 BDT	-0.103	-0.796	-0.247	**<0.001**	0.009	-0.502	0.745	0.703	0.027	-0.285	1.022	0.269
>30000 BDT	Reference				Reference				Reference			
**The person responsible for buying groceries**												
Father	-0.002	-0.501	0.474	0.957	-0.021	-1.465	0.745	0.523	-0.050	-2.024	0.293	0.143
Mother	-0.009	-0.618	0.479	0.804	0.026	-0.722	1.762	0.412	-0.021	-1.713	0.889	0.535
Other’s	Reference				Reference				Reference			
**Involvement in food preparation**												
Yes, daily	-0.050	-0.519	-0.029	**0.028**	0.034	-0.049	1.062	0.074	0.024	-0.227	0.938	0.231
No, never	-0.099	-1.142	-0.451	**<0.001**	-0.007	-0.945	0.629	0.694	0.070	0.686	2.336	**<0.001**
Yes, once a week	-0.031	-0.689	0.107	0.152	0.075	0.991	2.795	**<0.001**	0.040	0.041	1.939	**0.041**
Yes, once a month	-0.023	-0.964	0.279	0.280	0.026	-0.366	2.450	0.147	0.017	-0.784	2.168	0.358
Sometimes	Reference				Reference				Reference			
**Experience of food poisoning**												
Yes	0.014	-0.138	0.283	0.498	0.013	-0.295	0.659	0.455	0.060	0.328	1.328	**0.001**
No	Reference				Reference				Reference			
**Knowledge score**	–				0.555	1.399	1.589	**<0.001**	0.008	-0.096	0.142	0.710
**Attitude score**	–				–				0.414	0.369	0.457	**<0.001**

Note: Multiple linear regression analysis was performed to examine the independent associations between socio-demographic characteristics and food safety knowledge, attitude, and practice scores. β = standardized regression coefficient; CI = confidence interval; LL = lower limit; UL = upper limit. Reference categories are indicated as “Reference.” Statistical significance was considered at p < 0.05. BDT = Bangladeshi Taka.

ᵃ Knowledge model: F (31, 2194) = 5.307, p < 0.001; R^2^ = 0.070, adjusted R^2^ = 0.057, indicating that the model explained 7.0% of the variance in knowledge scores; Cohen’s f^2^= 0.075.

ᵇ Attitude model: F (32, 2193) = 35.44, p < 0.001; R^2^ = 0.341, adjusted R^2^ = 0.331, indicating that the model explained 34.1% of the variance in attitude scores; Cohen’s f^2^ = 0.517.

ᶜ Practice model: F (33, 2192) = 24.95, p < 0.001; R^2^ = 0.273, adjusted R^2^= 0.262, indicating that the model explained 27.3% of the variance in practice scores; Cohen’s f^2^ = 0.376.

The model that predicted Food Safety Attitudes was more explanatory, accounting for 34.1% of the variation (Adjusted R^2^ = 0.341). Consistent with the descriptive findings showing a borderline positive attitude, boys (β = 0.049, *p* = 0.007) and Class 9 students (β = 0.065, *p* = 0.001) demonstrated more encouraging attitudes toward food safety. Being involved in food preparation once a week was positively and significantly associated with better attitudes (β = 0.075, *p* < 0.001). Negative associations were found with certain educational and occupational statuses of parents: having a father with primary education and a mother with secondary education were both linked to less favorable attitudes. Additionally, having a mother whose occupation was categorized as business was associated with a less favorable food safety attitude score.

The food safety practices model explained 27.3% of the variance (Adjusted R^2^ = 0.273). In line with the descriptive results showing generally poor compliance with recommended practices, several strong positive predictors emerged: boys displayed significantly better practices (β = 0.134, *p* < 0.001), as did students attending government schools (β = 0.160, *p* < 0.001). Students whose fathers worked in agriculture or as laborers (e.g., farmers, fishermen, and laborers) also showed significantly better practice scores (β = 0.109, *p* = 0.002). Furthermore, both never and once weekly involvement in food preparation (compared to daily or once a month involvement) was associated with better practices. However, all categories of paternal education (from Primary to Higher Secondary) were negatively associated with food safety practices when compared to students whose fathers had no formal education, suggesting a complex relationship between paternal education and student practices. The regression analysis also highlighted a strong direct link between the three components, indicating that they are highly interdependent. Knowledge was found to be strongly associated with more favorable attitudes (β = 0.555, *p* < 0.001), and favorable attitude scores were significantly linked to better Practices (β = 0.414, *p* < 0.001).

### Division‐wise distribution of food safety KAP

**Fig 3:**
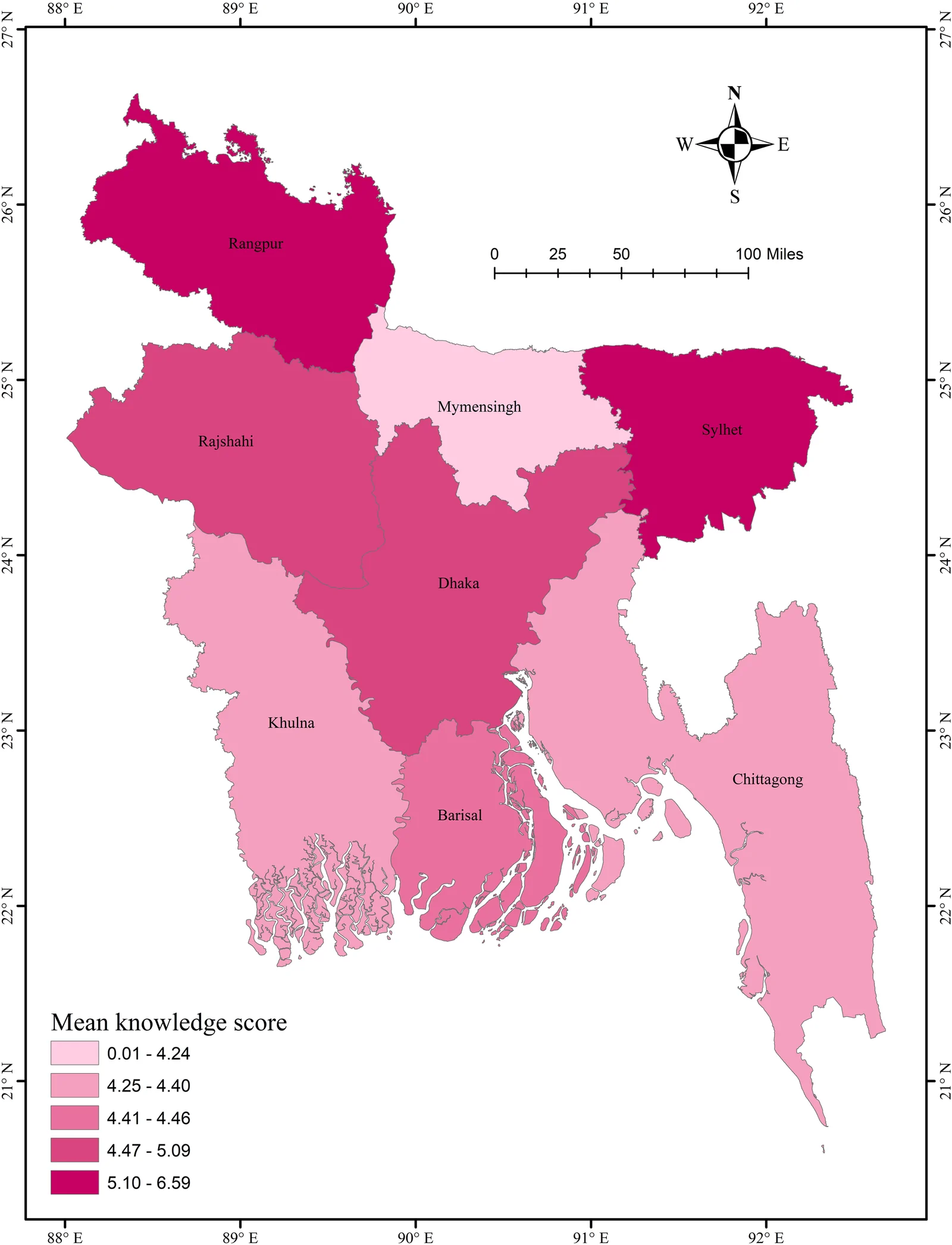
Division-wise food safety knowledge among school students in Bangladesh. The shape file for the GIS data is available at https://simplemaps.com/gis/country/bd.

**Fig 4:**
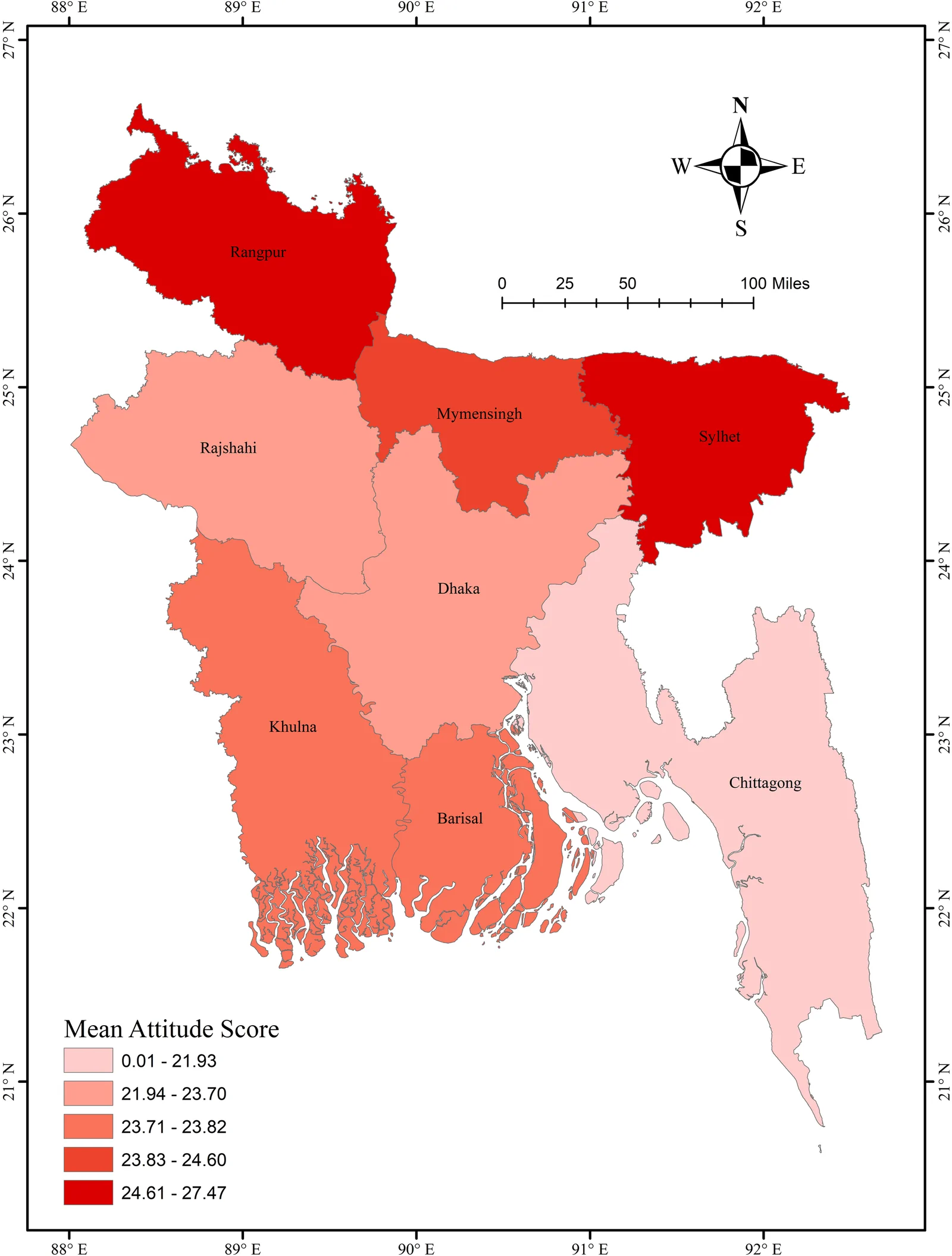
Division-wise food safety attitudes among school students in Bangladesh. The shape file for the GIS data is available at https://simplemaps.com/gis/country/bd.

**Fig 5:**
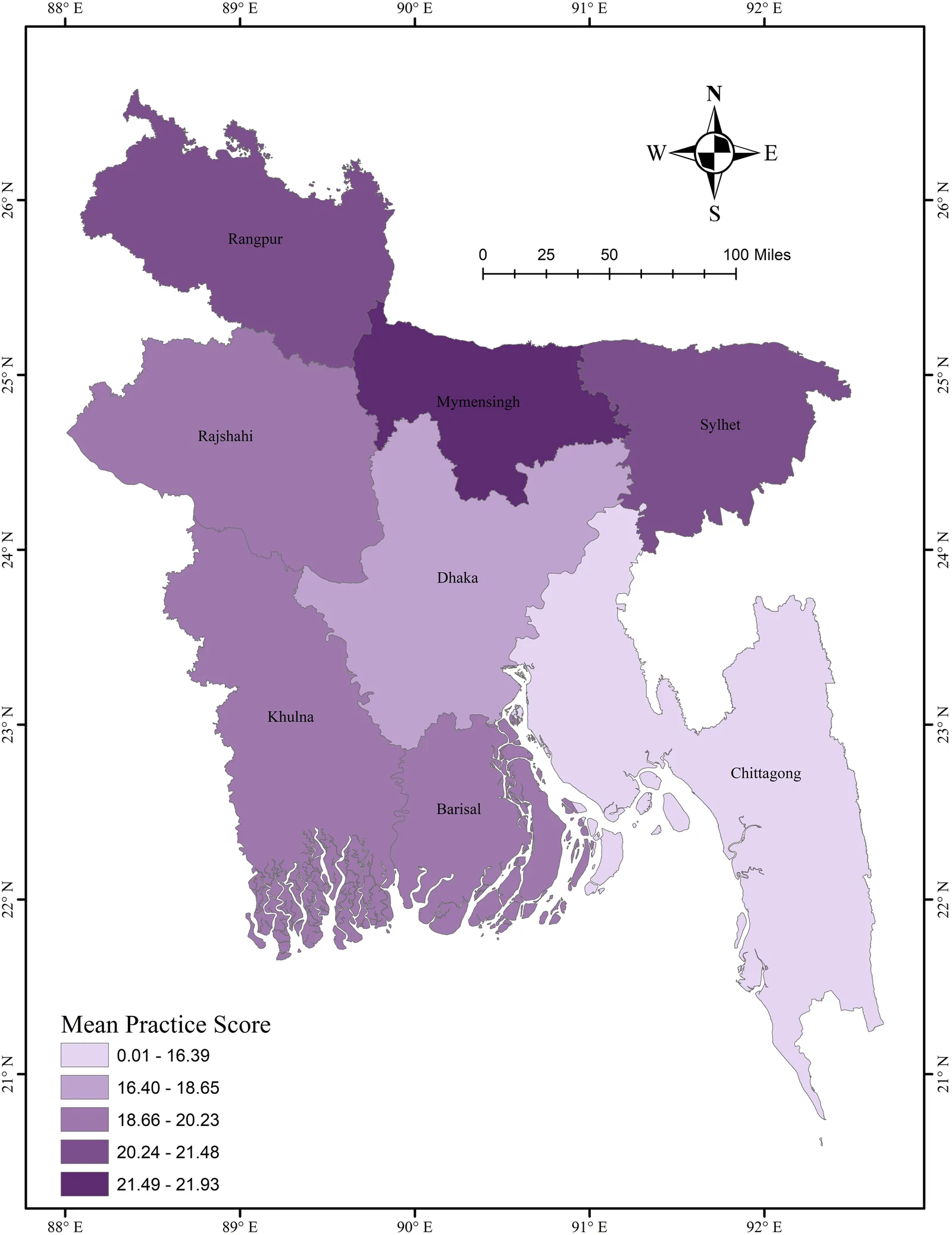
Division-wise food safety practices among school students in Bangladesh. The shape file for the GIS data is available at https://simplemaps.com/gis/country/bd.

**Fig 6:**
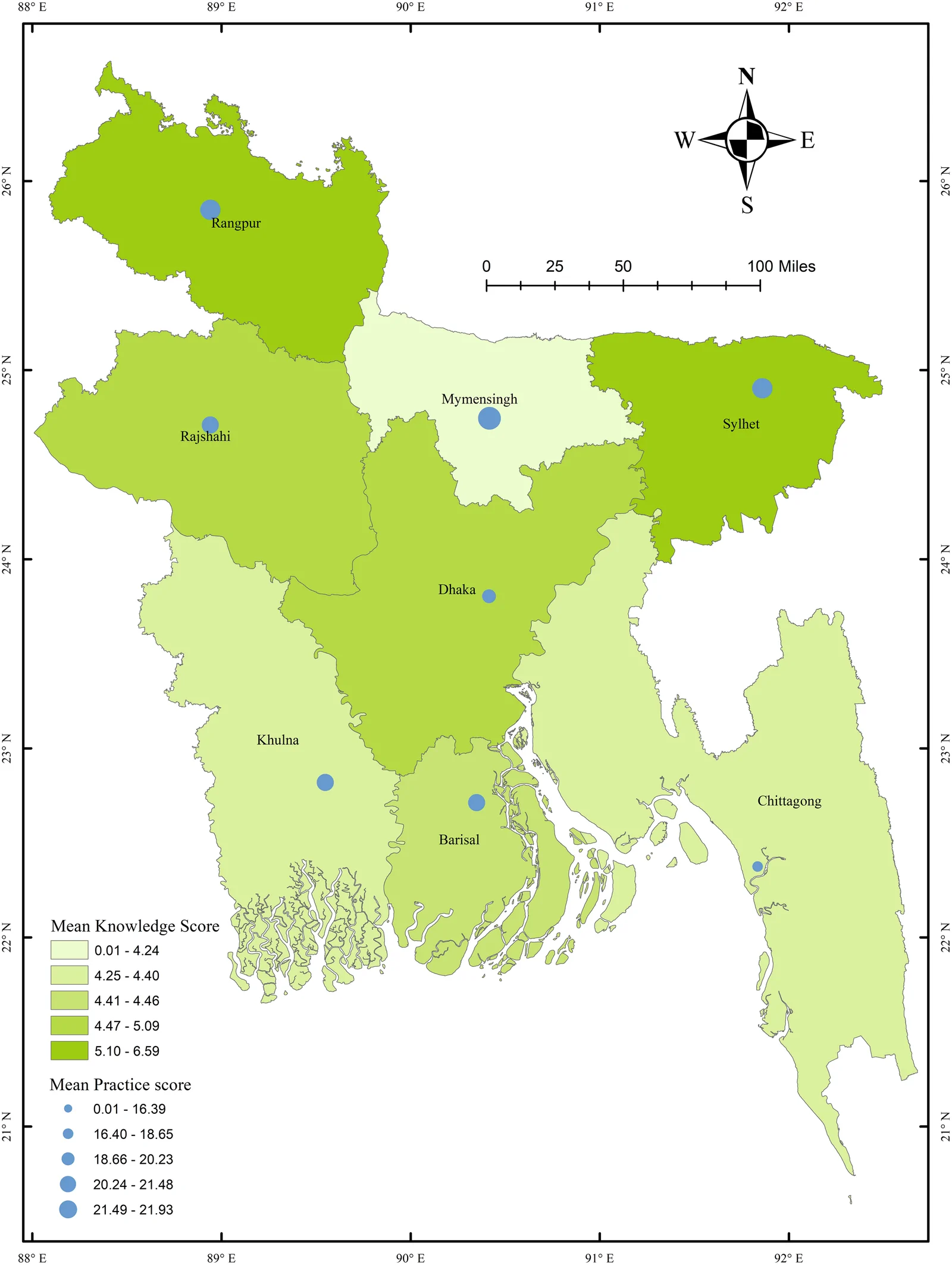
Division-wise food safety knowledge and practices among school students in Bangladesh. The shape file for the GIS data is available at https://simplemaps.com/gis/country/bd.

**Fig 7:**
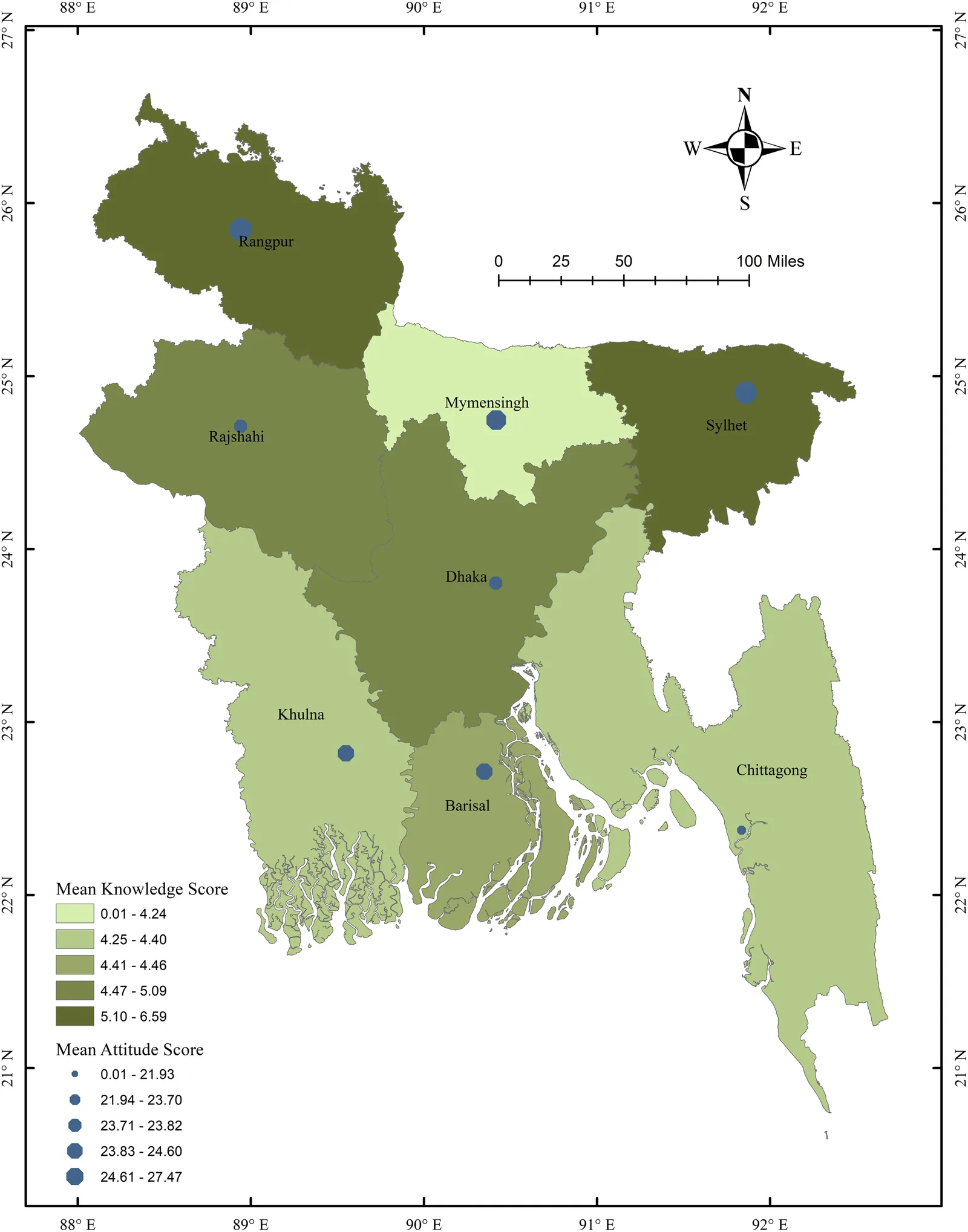
Division-wise food safety knowledge and attitudes among school students in Bangladesh. The shape file for the GIS data is available at https://simplemaps.com/gis/country/bd.

**Fig 8:**
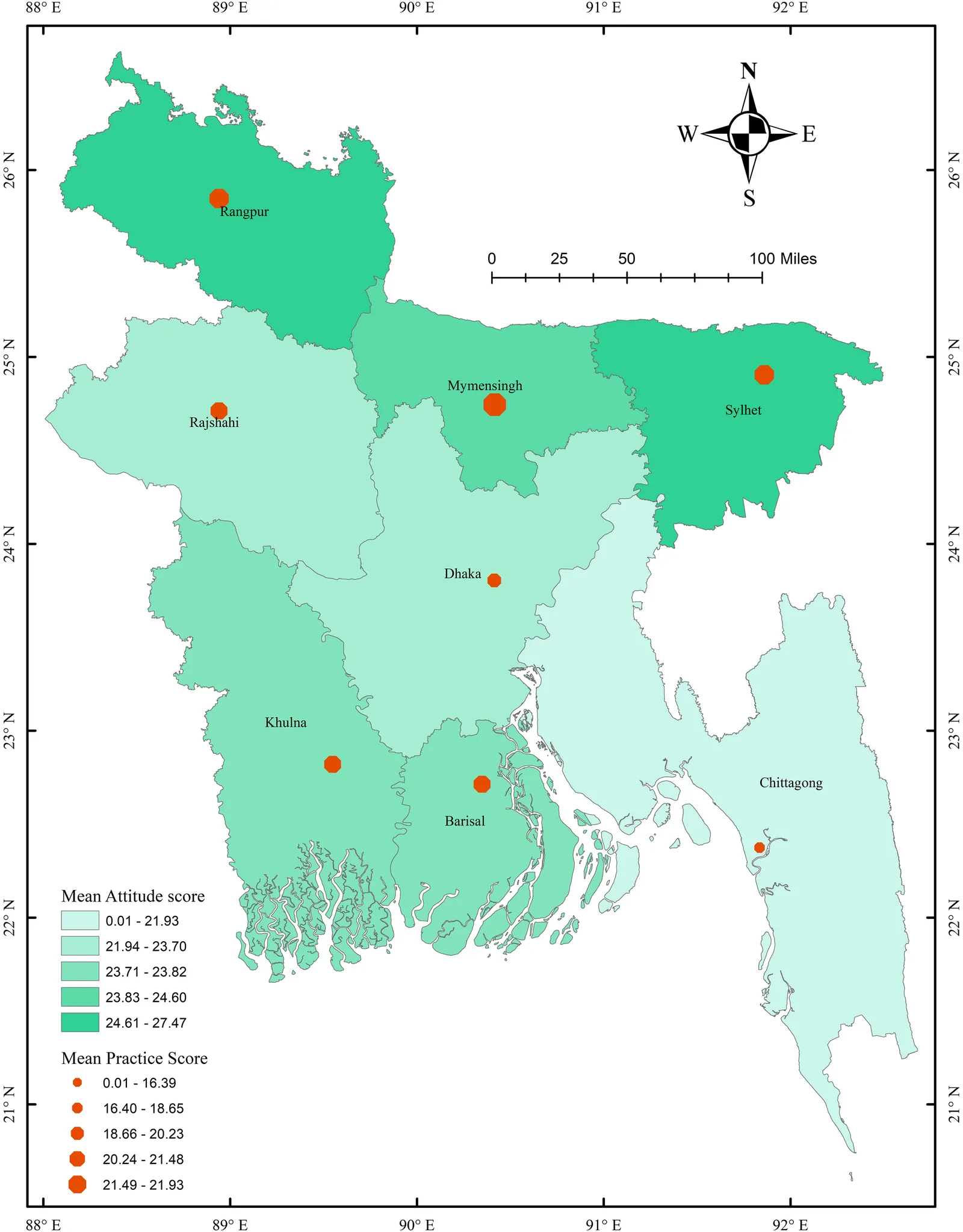
Division-wise food safety attitudes and practices among school students in Bangladesh. The shape file for the GIS data is available at https://simplemaps.com/gis/country/bd.

Further details of the literacy rate in terms of KAPs by division can be found in Fig 3, Fig 4, Fig 5.There existed substantial regional differences in food safety KAP among divisions (H = 239.675, p < 0.001 in knowledge, H = 125.700, p < 0.001 in attitudes, and H = 118.311, p < 0.001 in practices). This study found that the Rangpur, Sylhet, Dhaka, and Rajshahi divisions had good knowledge of food safety compared to the other divisions. Rangpur, Sylhet, and Mymensingh perceived higher food safety attitudes, while with respect to food safety practices, Mymensingh was the most consistent, followed by Rangpur and Sylhet as compared to other divisions. The associations between food safety knowledge and attitudes, knowledge and practices, and attitudes and practices are shown in Fig 6, Fig 7, Fig 8. At the division level, regions with more favorable food safety attitudes generally demonstrated better food safety practices. However, no significant correlations were observed between division-level average knowledge scores and either attitude or practice scores. These findings differ from the individual-level regression results (**Table 6**), where knowledge was positively associated with attitudes and attitudes were positively associated with practices. This suggests that relationships observed among individuals may not necessarily be reflected in aggregated regional patterns.

## Discussion

The objective of this study was to investigate the food safety KAP of selected secondary school students in Bangladesh. Moderate levels of knowledge, borderline positive attitudes, and poor food safety practices were observed among the respondents. Although some hygienic data were adequate, most misconceptions toward food hygiene and the level of practices towards food handlers remained inappropriate. These results highlight the importance of more active food safety education programs for adolescents in the school setting. Importantly, the coexistence of moderate knowledge and poor practices suggests that awareness alone is insufficient to drive behavioral change in this population.

The rate of correct answers indicated generally low food safety knowledge among students. This finding is consistent with studies from Brazil and USA, which also reported inadequate food safety knowledge among school students [13,49]. Students were relatively aware of some of the basic hygiene practices, such as handwashing before eating and refraining from preparation of food while being sick (which is also a positive outcome and can be attributed to public campaigns), similar to studies conducted in India and Namibia [50,51]. However, their knowledge of the safe storage of raw meat and ready-to-eat foods revealed a gap regarding the understanding of cross-contamination and temperature, consistent with findings reported in Lebanon and Serbia [52,53]. Poor understanding of leftover storage and reheating was found, suggesting a lack of awareness of bacterial growth and reheating safety, as previously reported among students in USA/UK/Lebanon [54]. The least knowledge domain was related to expired food, where respondents demonstrated poor understanding of labelling and the dangers of eating food that passed their expiry date, which is consistent with [55]. Taken together, these results indicate that although Bangladeshi students understand the elements of basic hygiene, they do not yet possess agricultural and food-handling expertise, supporting broader evidence that awareness does not automatically lead to high levels of food safety literacy. This disconnect suggests that current food safety knowledge is largely theoretical rather than actionable, limiting its translation into safe everyday practices. Hence, pragmatic education based on experiences in school health programs is required.

The overall response pattern showed marginally positive attitudes towards food safety, which implied that the students had a general desire to practice hygiene/risk avoidance, but there were common misconceptions among them in certain areas. In lines with the findings of a study in Kenya, where students had positive attitudes towards hygiene while lacking important knowledge about contamination and risks related to food labelling [56], Bangladeshi students appeared ambivalent. The majority of respondents expressed a high level of positive attitude toward crucial hygiene behaviors, such as acknowledging the necessity of undergoing a food hygiene course and washing hands before cooking. However, there were many myths in the community, especially about food additives being safe and that it was okay to eat expired edible items if they looked good. These findings are consistent with those reported in Cyprus, where students underestimated the health risks of additives and chemical preservatives [57]. The continuation of such misconceptions emphasizes a knowledge–attitude gap in food safety consciousness, indicating that students’ overall preference for hygiene is not directly linked to scientifically correct risk perception. This misalignment suggests that attitudes are shaped more by cultural beliefs and heuristics (e.g., appearance-based judgments of food safety) than by scientific understanding, which may partly explain why improved knowledge did not translate into safer behavior. Taken together, these elements underscore the necessity of targeted educational interventions tailored to modern food safety concerns (e.g., additives, labeling, and use-by dates) rather than simply teaching general hygiene practices.

Our findings indicated poor compliance of students with the recommended food safety measures, illustrating a wide discrepancy between knowledge, attitudes, and actual practice. Consistent with evidence from a systematic review, the respondents in this sample reported poor adherence to practicing safe food handling practices and storage food hygiene practices [58]. The majority of students only occasionally reheated food before consumption and commonly used sensory indicators (e.g., smell or appearance) to determine if food was safe for consumption, demonstrating potentially unsafe practices that have also been reported among USA/UK/Lebanese students [54]. Poor storage practices, wherein raw and cooked foods were left together or stored and allowed to thaw at room temperature before cooking, also indicated a lack of knowledge about the risks of cross-contamination posed and is comparable with results from Indonesia and the United Arab Emirates, where similar poor handling was reported [55,59]. Additionally, preventive activities (e.g., checking or discarding expired foods, avoiding products that may contain substances that are unknown additives) were seldom deployed secondary to the behavior of interest, and was consistent with prior research findings linking low-risk perceptions to poor behavioral adherence [55]. A low frequency of handwashing, as well as insufficient rinsing of the fruit, points to ongoing barriers to hygiene despite a modest level of knowledge and indicates that cognitive awareness in itself may not necessarily translate into safe practices. In general, our findings are in line with international evidence, as they show that most adolescents do not know how to put into practice their acquired food safety knowledge and attitudes; therefore, there is an urgent requirement for behavior-oriented educational learning strategies in schools at a global scale to improve practical food safety practices.

In the regression analysis, sociodemographic characteristics that were significantly associated with KAP toward food safety among students were identified. Consistency in the importance of sex difference was observed with boys having statistically higher levels than girls for each of the components: KAP, reflecting previous work conducted in Sri Lanaka that reported male students to have better food safety knowledge [60]. School grade was also a determinant, with grade 9 students having significantly better food safety awareness and attitudes than grade 10 students. This difference may reflect variations in curriculum exposure and school engagement between grade levels; however, the underlying reasons remain unclear and require further investigation. Parental education, particularly fathers’ lower education levels were inversely associated with students’ KAP, and we found support for previous research that claimed parent educational level is related to household food safety norms [60]. Notably, students from rural areas had higher knowledge scores than their counterparts from urban areas, similar to a study conducted in Indonesia [61]. Family income was also a strong predictor; it seems that students with lower family income had worse food safety knowledge, probably because of low access to health information or food safety sources. Involvement in food preparation was indicative of nuanced relationships: Moderate involvement (e.g., once a week) was associated with more positive attitudes and practices (although daily and no involvement were both negatively related), but both high engagement and no engagement were related to lower knowledge, suggesting that either extreme can reduce learning opportunities. Overall, these findings suggest a non-linear relationship between exposure and learning, where neither passive observation nor excessive routine involvement guarantees effective knowledge internalization or behavioral improvement.

The study also found that knowledge was positively correlated with attitude and attitude was significantly associated with practice. This order of association accords with the knowledge, attitude, and–practice framework in which knowledge affects perception and practice [62]. Yet, our findings of the weak to moderate relationship between knowledge and practice in the present study show that provision of knowledge alone may not be enough to change behavior. This decoupling between knowledge and practice suggests that behavioral execution is likely mediated by psychological and environmental factors rather than knowledge alone. Successful interventions need to work across the cognitive-behavioral divide and include experiential learning, behavioral modeling, and reinforcement methodologies. To truly understand why this gap exists, attention must be paid to the real-world environments where these students live and learn. Knowing a rule does not mean a student has the power or the resources to follow it. For example, a student might know that thorough handwashing is critical, but if their school lacks running water, soap, or clean facilities, practicing that knowledge becomes nearly impossible. Similarly, at home, teenagers have very little control over how food is handled. Traditional family habits, cultural norms, and parental decisions dictate how groceries are bought, stored, and cooked. Even if a student recognizes that storing raw meat next to cooked food is risky, they usually lack the authority to change how the household refrigerator is organized. Because of these barriers, textbook knowledge remains purely theoretical. Future health programs must look beyond the classroom and focus on fixing school infrastructure while actively involving parents, creating environments where students can actually put their knowledge into action.

The region-wise breakdown indicated significant differences in the KAP status of students in Bangladesh, which clearly reflects that regional or socio-cultural settings have a significant impact on food safety behavior. Higher knowledge levels observed in Rangpur, Sylhet, Dhaka, and Rajshahi may reflect variations in access to educational resources, exposure to health information, and differences in school-based learning environments across regions. It is likely that the higher levels of food safety attitudes found in Rangpur, Sylhet, and Mymensingh may be influenced by regional differences in cultural practices and household norms for preparing food, where cooking relatively more with family members and community health programs may help develop better hygiene attitudes. Evidence of better adherence to safe food practices among Mymensingh, Rangpur, and Sylhet students is in line with the idea that local practical know-how and reinforcement can help cultivate safer practices, despite having low formal knowledge. Nonetheless, better knowledge was not correlated with improved practices, highlighting an already reported problem in food safety education the knowledge behavior gap. This gap indicates that exposure to evidence alone may not lead to safety practice in the absence of behavioral reinforcement, motivation, and environmental support. This pattern reinforces the notion that structural and contextual constraints, rather than individual knowledge deficits alone, may be the primary drivers of unsafe practices in this setting. The GIS-identified spatial clustering further reveals that these constraints are highly regionalized, likely reflecting deeper socioeconomic and environmental disparities across Bangladesh. For instance, lower KAP clusters may stem from localized challenges, such as poorer water and sanitation infrastructure, lower average household incomes, or limited access to national media campaigns in remote divisions. Conversely, high-performing clusters suggest areas benefiting from active regional non-governmental organization programs or stronger local school monitoring. This spatial heterogeneity demonstrates that food safety risks are not uniform; instead of broad national strategies, policymakers must use these GIS insights to deploy targeted financial and educational resources to the highest-risk divisions. The relationship between favorable attitudes and better practices was significant, suggesting that attitude plays a mediating role between knowledge and practice, which supports the KAP model for public health.

### Implications of the study

To improve food safety KAP efficaciously, it is critical to design tailored knowledge modules for expired foods, food storage, and safe disposal of leftovers using a scenario-based approach to reinforce domain-specific learning where deficiencies exist [63–65]. Activities should also be designed to target misconceptions (e.g., the safety of additives and misinterpretations of dating) by bringing attitudes in line with perceived risk and debunking inaccurate beliefs through specifically tailored educational materials [66,67]. Conducting practical exercises in food safety training and the availability of safety tools can still strengthen the learning process, underlining the significance of structured practice opportunities, as demonstrated by previous evaluations [68,69]. Further, the observation by the study that determinants of KAP scores, such as demographic characteristics including sex, parental education, household income, and participation in food preparation, are among the predictors of resource planning for an effective high impact. Intervention programs should focus on these target groups of the most vulnerable, for example, students from families with lower incomes or lower levels of parental education [70–72]. In addition, because food safety habits are fundamentally formed at home, interventions must extend beyond the classroom to actively engage parents and caretakers [26,73]. Since parents control household budgets, grocery choices, and kitchen routines, school-based lessons will have a limited impact unless reinforced by parental behavior. To bridge this gap, school health programs should integrate family-centered take-home modules or shared assignments that encourage parents to model and co-create safe food environments at home. Importantly, based on the observed regional variation in KAP levels, particularly the GIS-identified spatial clustering of low KAP areas, implementation strategies should include targeted school-level interventions in high-risk divisions. These should involve structured food safety training programs for school food handlers (e.g., canteen staff and vendors) focusing on hygienic food preparation, safe storage, and contamination prevention practices. Furthermore, educational institutions should adopt formal food safety guidelines or policies, including routine monitoring of school canteens, mandatory hygiene standards for food vendors, and periodic inspection systems to ensure compliance with basic food safety requirements. These institutional measures are necessary to translate knowledge and awareness into safe food environments within schools. Finally, the use of GIS generates a strong location-based evidence base, exhibiting spatial differentials in food safety KAP scores. This allows policymakers to move away from generic national-level campaigns and instead implement geographically targeted school and community interventions in high-risk areas identified through spatial analysis, ensuring more efficient resource allocation [74,75]. It is necessary to institutionalize these behaviors through the integration of food safety education into the official national secondary curriculum, supported by continuous training of school food handlers and enforcement of school-level food safety policies, to ensure sustained improvements in food safety practices and reduction of foodborne risks among students [70,71].

### Limitations

Despite the relatively large sample (2226 students) and nationwide coverage, this study has some limitations that should be considered when interpreting the results. First, the cross-sectional design limits causal inference. The data provide only a single-time “snapshot” of food safety KAP, meaning that temporal relationships and directionality (e.g., whether knowledge influences practice or vice versa) cannot be established. Therefore, observed associations between sociodemographic factors and KAP scores should be interpreted as correlational rather than causal. Second, the study relied on self-reported measures of food safety practices, which are subject to recall bias and social desirability bias. Participants may have over-reported desirable behaviors (e.g., handwashing, safe food handling) and under-reported unsafe practices. As a result, the actual prevalence of unsafe food handling behaviors may be higher than what is reported in this study. Future studies should incorporate direct observation or mixed-method approaches to validate self-reported behavioral data. Third, even though the study was countrywide, the sample was drawn using the standard method, and thus may not be fully representative of an extremely varied environment in secondary schools within all remote or otherwise highly diverse socioeconomic areas within Bangladesh. Finally, although the questionnaire is a standard instrument, it does not reveal how deep or qualitatively deep perceptions are held by students with respect to safe food practices.

## Conclusion

The present study is the first GIS-based large-scale cross-sectional assessment on food safety KAP among secondary level students in Bangladesh. The results demonstrated significant discrepancies between the domains of food safety: students showed moderate levels of knowledge and favorable attitudes, but findings were not consistently reflected in safe self-reported behaviors; the result that awareness is necessary but still insufficient to change their behavior during this period. In addition, an inverse association was found between several sociodemographic determinants (lower maternal education, lower household income, and less involvement in preparing food at home) and inadequate KAP. The novel approach to GIS offers compelling evidence of spatial inequalities, demonstrating that food safety problems seem to be concentrated and not uniformly distributed. It underscores the urgent need to include practical and behavior-focused food safety education in the secondary school curriculum nationwide to tailor any geographically specific public health intervention. These efforts will build an infrastructure for literacy in food safety, generate sustained behavior changes, and help decrease the burden of foodborne illness at a national level.

## Supporting information

S1 Data Dataset for the present study. (XLSX)
